# IVT-seq reveals extreme bias in RNA sequencing

**DOI:** 10.1186/gb-2014-15-6-r86

**Published:** 2014-06-30

**Authors:** Nicholas F Lahens, Ibrahim Halil Kavakli, Ray Zhang, Katharina Hayer, Michael B Black, Hannah Dueck, Angel Pizarro, Junhyong Kim, Rafael Irizarry, Russell S Thomas, Gregory R Grant, John B Hogenesch

**Affiliations:** 1 Department of Pharmacology, University of Pennsylvania Perelman School of Medicine, Philadelphia, PA, USA; 2 Department of Chemical and Biological Engineering, Koc University, Istanbul, Turkey; 3 Department of Molecular Biology and Genetics, Koc University, Istanbul, Turkey; 4 The Institute for Translational Medicine and Therapeutics, University of Pennsylvania Perelman School of Medicine, Philadelphia, PA, USA; 5 The Hamner Institutes for Health Sciences, Research Triangle Park, NC, USA; 6 Department of Biology, University of Pennsylvania, Philadelphia, PA, USA; 7 Amazon Web Services, Herndon, VA, USA; 8 Department of Biostatistics, Johns Hopkins University Bloomberg School of Public Health, Baltimore, MD, USA; 9 Department of Genetics, University of Pennsylvania Perelman School of Medicine, Philadelphia, PA, USA

## Abstract

**Background:**

RNA-seq is a powerful technique for identifying and quantifying transcription and splicing events, both known and novel. However, given its recent development and the proliferation of library construction methods, understanding the bias it introduces is incomplete but critical to realizing its value.

**Results:**

We present a method, *in vitro* transcription sequencing (IVT-seq), for identifying and assessing the technical biases in RNA-seq library generation and sequencing at scale. We created a pool of over 1,000 *in vitro* transcribed RNAs from a full-length human cDNA library and sequenced them with polyA and total RNA-seq, the most common protocols. Because each cDNA is full length, and we show *in vitro* transcription is incredibly processive, each base in each transcript should be equivalently represented. However, with common RNA-seq applications and platforms, we find 50% of transcripts have more than two-fold and 10% have more than 10-fold differences in within-transcript sequence coverage. We also find greater than 6% of transcripts have regions of dramatically unpredictable sequencing coverage between samples, confounding accurate determination of their expression. We use a combination of experimental and computational approaches to show rRNA depletion is responsible for the most significant variability in coverage, and several sequence determinants also strongly influence representation.

**Conclusions:**

These results show the utility of IVT-seq for promoting better understanding of bias introduced by RNA-seq. We find rRNA depletion is responsible for substantial, unappreciated biases in coverage introduced during library preparation. These biases suggest exon-level expression analysis may be inadvisable, and we recommend caution when interpreting RNA-seq results.

## Background

High-throughput sequencing of RNA (RNA-seq) is a powerful suite of techniques to understand transcriptional regulation. Using RNA-seq, not only can we perform traditional differential gene expression analysis with better resolution, we can now comprehensively study alternative splicing, RNA editing and allele-specific expression, and identify novel transcripts, both coding and non-coding RNAs [1-3]. In contrast to the more established microarray-based RNA expression analysis, the flexibility of RNA-seq has allowed for the development of many different protocols aimed at different goals (for example, gene expression of polyadenylated (polyA) transcripts, small RNA sequencing, and total RNA sequencing). However, this same flexibility has the potential for complex technical bias, because different methods are routinely employed in RNA isolation, size selection, fragmentation, conversion to cDNA, amplification and, finally, sequencing [4-7]. While progress has been made in generating and analyzing RNA-seq data, we understand comparatively little about the technical biases the various protocols introduce. Understanding these biases is critical to differential analysis, to avoiding experimental artifacts (for example, in characterizing RNA editing), and to realizing the full potential of this powerful technology.

Previous efforts at understanding bias identified several contributing sources, including GC-content and PCR enrichment [8,9], priming of reverse transcription by random hexamers [10], read errors introduced during the sequencing-by-synthesis reaction [11], and bias introduced by various methods of rRNA subtraction [7]. Studies that revealed these sources of bias typically used computational methods on existing sequencing data to assess the performance of various sequencing technologies and library protocols. One downside to this approach is that it can be difficult to know whether anomalies in coverage are natural, or are due to technical artifacts. For example, nearly every RNA-seq study has differences in intra-exonal coverage, which could arise from naturally occurring splice variants sharing part of an exon, or could be due to technical error in library construction or sequencing.

Given that researchers are continually developing new sequencing methodologies and library generation protocols [12], we need a means for assessing the technical biases introduced by each new iteration in technology. One attractive alternative is to generate libraries from RNA that has been *in vitro* transcribed (IVT) from cDNA clones, where the nucleotide sequence at every base is known, the splicing pattern established and inviolate, and the expression level is known to be uniform across the transcript. Thus, any observed biases in coverage or expression must be technical rather than biological. This is the experimental equivalent of simulated data that computational researchers commonly use to develop and assess alignment algorithms [13-15]. Jiang and colleagues used a similar approach with 96 synthetic sequences derived from *Bacillus subtilis* or the deep-sea vent microbe *Methanocaldococcus jannaschii* genomes [16], organisms that do not have RNA splicing or polyadenylation. The focus of that work, though, was creating a useful set of standards that could be used in downstream analysis, not exploring library construction bias in a comprehensive set of complex mammalian samples.

Here we present and apply IVT-seq at scale to better understand bias introduced by RNA-seq. In brief, individual plasmids were produced, pooled, and subjected to *in vitro* transcription. Next, this RNA was mixed with complex mouse total RNA at various concentrations, and sequenced using the two most common RNA-seq protocols, polyA-seq or total RNA-seq, on the Illumina platform. We found coverage bias in most IVT transcripts, with over 50% showing greater than two-fold changes in within-transcript coverage and 10% having more than 10-fold differences attributable to library preparation and sequencing. Additionally, we found greater than 6% of IVT transcripts contained regions of high, unpredictable sequencing coverage, which varied significantly between samples. These biases were highly reproducible between replicates and suggest that exon-level quantification may be inadvisable. Furthermore, we created sequencing libraries from the original plasmid templates and using several different RNA selection methods (rRNA depletion, polyA selection, and no selection). We found that both rRNA depletion and polyA selection are responsible for a significant portion of this coverage bias, and computational analysis showed that poorly represented regions of transcripts are associated with low complexity sequences. Taken together, these results show the utility of the IVT-seq method for characterizing and identifying the sources of coverage bias in sequencing technologies.

## Results and discussion

### IVT-seq library preparation and sequencing

**Figure 1:**
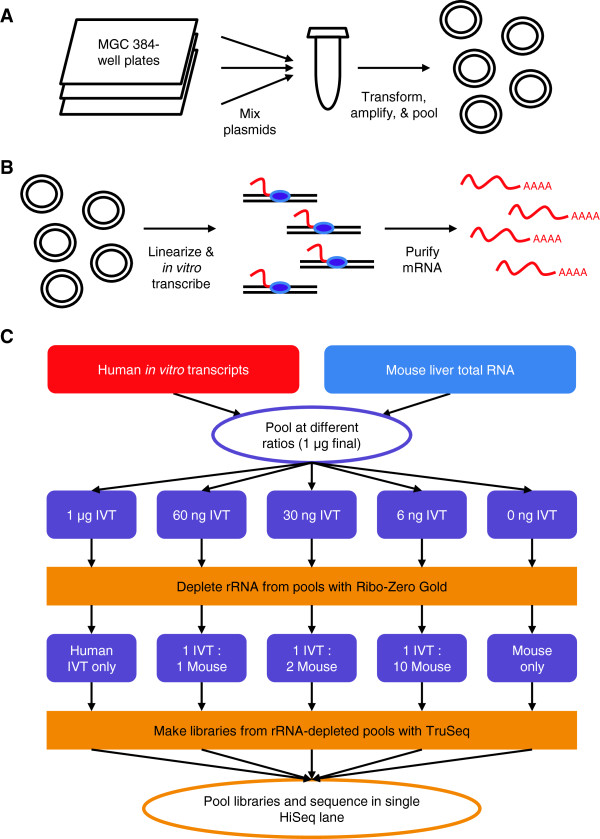
**Construction of IVT-seq libraries. (A)** Preparation of a pool of 1,062 human cDNA plasmids. Contents of three 384-well plates containing MGC plasmids were pooled together. Pool was amplified via transformation in *Escherichia coli*, and resulting clones were purified and re-pooled. **(B)** Generation of IVT transcripts. Pool of MGC plasmids was linearized and used as a template for an *in vitro* transcription reaction. Enzymes and unincorporated nucleotides were purified, leaving pool of polyA transcripts. **(C)** Creation of IVT-seq libraries. Listed quantities of IVT RNA were mixed with mouse liver total RNA to create six pools with final RNA quantities of 1 μg. Ribosomal RNA was depleted from these pools using the Ribo-Zero Gold kit. IVT RNA and mouse RNA are now present in pools at the listed ratios, following depletion of rRNA from mouse total RNA. These pools were used to generate RNA-seq libraries using Illumina’s TruSeq kit/protocol. This entire process was performed in duplicate. Replicate libraries were pooled separately and sequenced in separate HiSeq 2000 lanes (two lanes total). IVT, *in vitro* transcribed; MGC, Mammalian Gene Collection.

To generate IVT-seq libraries (for full details, please see the *Materials and methods* section), we produced individual glycerol stocks each harboring a single, human, fully sequenced plasmid from the Mammalian Gene Collection (MGC) [17]. Next, we extracted the plasmid DNA and plated it at 50 ng per well in 384-well plates. We mixed the contents of three 384-well plates containing a total of 1,062 cDNA clones (Additional file 1), transformed this mixture into bacteria, and plated the bacteria as single colonies. Following an overnight incubation, we scraped these plates, amplified the bacteria for a few hours in liquid culture, and purified plasmids from the bacteria as a pool (Figure 1A). Next, we linearized the plasmids, and used SP6 polymerase to drive *in vitro* transcription of the cloned cDNA sequences (Figure 1B). Following a DNase I treatment to remove the DNA template and RNA purification, we were left with a pool of 1,062 different human RNAs derived from fully sequenced plasmids.

To approximate what happens in a total RNA-seq reaction, we subjected this IVT RNA to rRNA depletion and then prepared libraries using the Illumina TruSeq protocol (Figure 1C, IVT only). To account for possible carrier effects, we also mixed the IVT RNA with various amounts of mouse total RNA derived from liver. The addition of the mouse RNA gave these samples greater diversity (transcripts from approximately 10,000 genes versus 1,062) and more closely resembled a real biological sample. Also, by adding background RNA from a different species (mouse) than the IVT RNA (human), we made it easier to differentiate between the IVT transcripts and mouse sequences during downstream analysis. Because the IVT RNA did not contain rRNA sequences whereas the mouse RNA did, the quantity of mouse RNA would be significantly reduced by the rRNA depletion step. To account for this, we mixed IVT and mouse RNA such that, following rRNA depletion, we would have final pools with IVT:mouse ratios of 1:1, 1:2, and 1:10. Finally, to account for mouse RNAs potentially mapping to the human reference genome and our IVT sequences, we prepared a pool consisting of mouse RNA alone. We pooled the resulting six libraries and sequenced them using an Illumina HiSeq 2000. We performed this entire process in duplicate.

### Mapping and coverage of IVT-seq data

Following sequencing and de-multiplexing, we aligned all of the data to the human reference genome (hg19) using the RNA-seq Unified Mapper (RUM) [14]. For all analyses, we only used data from reads uniquely mapped to the reference, excluding all multi-mappers (data contained in RUM_Unique and RUM_Unique.cov files). Of the 1,062 original IVT transcripts, we found 11 aligned to multiple genomic loci, while 88 aligned to overlapping loci. To avoid any confounding effects in our analyses, we filtered those transcripts from all analyses, leaving us with 963, non-overlapping, uniquely-aligned IVT transcripts. We saw excellent correlation in expression levels between replicates (transcript-level R^2^ between replicates >0.95; Additional file 2: Figure S1A). Secondly, at least 90% of the 963 IVT transcripts were expressed with fragments per kilobase of exon per million mapped reads (FPKM) values ≥5 in all IVT-seq datasets, except mouse only (Table 1). In the IVT-only samples, over 80% of the IVT sequences were expressed above 100 FPKM (Additional file 2: Figure S1B). Because we prepared the MGC plasmids and IVT transcripts as pools, it is likely that the IVT transcripts showing low or zero coverage were initially present at low plasmid concentrations prior to the transformation and IVT steps. Using the IVT-seq technique, we were able to specifically detect the vast majority of the human IVT transcripts with high coverage in both the absence and presence of the background mouse RNA.

**Table 1:** Detection of source cDNA sequences in IVT-seq

**Total number of cDNA clones:**	**963**	
	**Replicate 1**	**Replicate 2**
Number of clones detected (FPKM ≥5):		
IVT only	869	870
1:1 Mix	877	876
1:2 Mix	886	883
1:10 Mix	896	892
Mouse only	278	271
PolyA selection	829	-
No selection	870	-
Plasmid library	924	-
Average, normalized^a^ depth of coverage for detected clones:		
IVT Only	76.09	80.22
1:1 Mix	75.15	75.06
1:2 Mix	65.79	69.40
1:10 Mix	37.50	47.46
Mouse only	01.58	02.42
PolyA selection	72.27	-
No selection	72.74	-
Plasmid library	42.08	-

^a^Average depth of coverage is normalized by the number of millions of fragments mapped to the human reference in each sample.

While we do see reads aligned to the human IVT transcripts in the mouse-only data, these transcripts collectively represent approximately 2% of reads (Table 1). Those transcripts with higher coverage are likely the result of mouse reads aligning to highly similar human sequences. We excluded these sequences from our analyses.

### Within-transcript variation in RNA-seq coverage of IVT transcripts

Consider first the IVT-only data. Given that these transcripts were generated from an IVT reaction using cDNA sequences, these data are unaffected by splicing or other post-transcriptional regulation. Thus, most regions of transcripts should be ‘expressed’ and present at similar levels. The exceptions would be repetitive sequences that map to multiple genome locations and may be poorly represented, and the ends of the cDNAs, which are subject to fragmentation bias. To account for this, we created a simulated dataset that models the fragmentation process and deviates from uniform data only by the randomness incurred by fragmentation. We generated two such datasets using the Benchmarker for Evaluating the Effectiveness of RNA-Seq Software (BEERS) [14]. The first dataset contained all of the IVT transcripts expressed at roughly the same level of expression (approximately 500 FPKM). For the second, we used FPKM values from the IVT-only samples as a seed, creating a simulated dataset with expression levels closely matching real data (Additional file 3: Figure S2). These datasets are referred to as simulated and quantity matched (QM)-simulated, respectively. The simulated data provide an ideal result, while the QM data allow us to control for any artifacts arising from expression level (for example, transcripts with lower expression may show more variability). Next, we aligned both simulated datasets using RUM, with the same parameters as for the biological data. Thus, both simulated datasets also serve as controls for any artifacts introduced by the alignment (for example, low coverage in repeat regions). For full details on the creation of simulated data, see the *Materials and methods* section.

**Figure 2:**
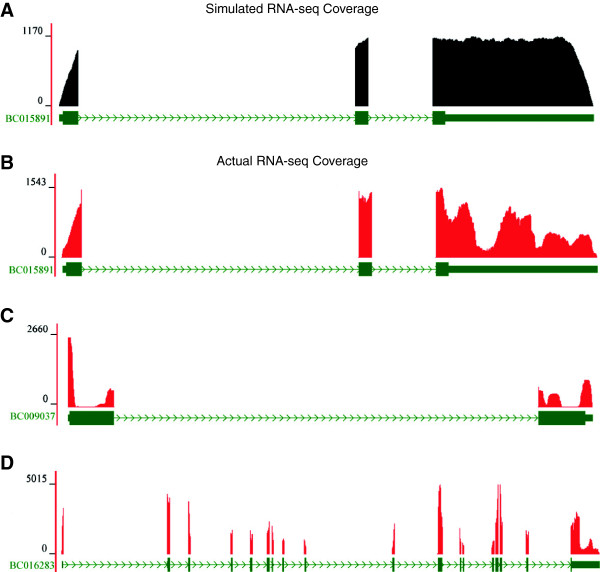
**Within-transcript variations in RNA-seq coverage. (A)** Simulated RNA-seq coverage for a representative IVT transcript (BC015891). RNA-seq coverage plot (black) is displayed according to the gene model (green), as it is mapped to the reference genome. Blocks correspond to exons and lines indicate introns. The chevrons within the intronic lines indicate the direction of transcription. Numbers on y-axis refer to RNA-seq read-depth at a given nucleotide position. **(B)** The actual RNA-seq coverage plot for BC015891 in the IVT-only sample. Representative coverage plots for the IVT transcripts **(C)** BC009037 and **(D)** BC016283 are displayed according to the same conventions used above. All transcripts are displayed in the 5ʹ to 3ʹ direction.

Using IVT data derived from the BC015891 transcript as a representative example, the ideal, theoretical coverage plot from the simulated data shows near-uniform coverage across the transcript’s entire length, with none of the extreme peaks and valleys characteristic of biological datasets (Figure 2A). However, our observed data showed a high degree of variability, with peaks and valleys within an exon (Figure 2B). Furthermore, these patterns were reproducible across our replicates (Additional file 4: Figure S3). We saw many other cases of extreme changes in coverage: over 50% of the IVT transcripts showed greater than two-fold changes in within-transcript coverage attributable to library preparation and sequencing (Table 2 and Additional file 5: Figure S4). For example, BC009037 showed sudden dips to extremely low levels of expression in both of its exons (Figure 2C). Both simulated datasets showed no such patterns, which indicates this coverage variability is not the result of alignment artifacts. Furthermore, the absence of this pattern in the QM-simulated data indicates these fold-change differences in coverage were not due to sampling noise introduced by transcripts with low or high coverage. In the case of BC016283, the peaks and valleys in coverage led to greater than five-fold differences in expression levels between exons (Figure 2D). Once again, these patterns were reproducible across replicates (Additional file 4: Figure S3). The SP6 polymerase cannot fall off and then re-attach at a later point in the transcript, leaving a region un-transcribed. Therefore, given that these patterns showed troughs followed by peaks, they cannot be the result of artifacts from *in vitro* transcription. Furthermore, we sequenced the IVT products directly and found the vast majority were transcribed with little to no bias. Taken together, these data suggest that these coverage patterns are primarily the result of technical biases introduced during library construction, rather than biology. These results are consistent with a previous study that used IVT RNA as standards in RNA-seq experiments [16], suggesting that our IVT-seq methodology is suitable for identifying technical variability in sequencing data.

**Table 2:** Fold-change differences in within-transcript coverage by library type

	**Number of IVT-transcripts with fold-change differences:**		
	**>2**	**>10**	**>100**
rRNA-depleted	713 (74.0%)	110 (11.4%)	17 (1.7%)
PolyA selection	678 (70.4%)	163 (16.9%)	7 (0.7%)
No selection	400 (41.5%)	31 (3.2%)	3 (0.3%)
Plasmid	189 (19.6%)	14 (1.5%)	3 (0.3%)
Simulated	0	0	0
QM-simulated	0	0	0

The plasmid data provides a measure of bias from library preparation/sequencing, while the no selection data accounts for potential artifacts from the *in vitro* transcription (IVT) step. To calculate the percentage of transcripts affected by bias due specifically to library preparation and sequencing, but not sequence or *in vitro* transcription artifacts, we performed the following calculation: rRNA depletion% - no selection% + plasmid%. So we found 74% – 41.5% + 19.6% = 52.1% of transcripts in the rRNA-depleted data have greater than two-fold difference in coverage, and 11.4% – 3.2% + 1.5% = 9.7% have greater than 10-fold difference in coverage. QM, quantity matched.

### Between-sample variation in RNA-seq coverage of IVT transcripts

**Figure 3:**
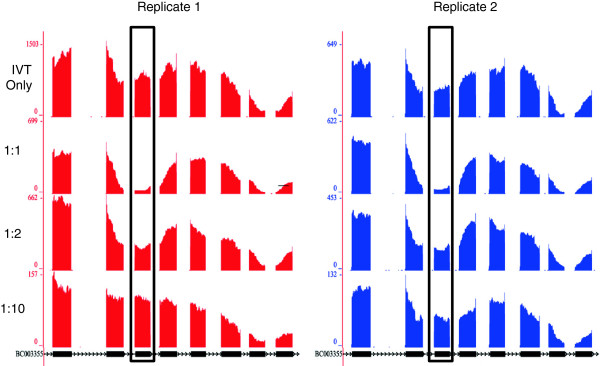
**Between-sample variations in RNA-seq coverage.** RNA-seq coverage plots across all samples for exons 4 to 11 of the IVT transcript BC003355. The black rectangles identify exon six, which shows extreme variability in coverage relative to the rest of the transcript when viewed across all of the samples. The ratio of IVT RNA to mouse RNA is listed to the left of each sample’s coverage plots. Coverage plots (red for first replicate; blue for second replicate) are displayed according to the gene model (black), as it is mapped to the reference genome. Blocks in the gene model correspond to exons and lines indicate introns. The chevrons within the intronic lines indicated the direction of transcription. Numbers on y-axes refer to RNA-seq read-depth at a given nucleotide position.

In addition to this variability within transcripts, we also found many transcript regions showing extreme variability in coverage across samples (Figure 3). For example, the sixth exon of BC003355 varied wildly relative to the remainder of the transcript across all IVT:mouse dilutions. Interestingly, the overall pattern of variation relative to the rest of the transcript across the dilutions was maintained between the replicates. Almost no reads in the mouse-only sample map to this transcript, which eliminates the possibility that this variability was due to incorrect alignment of mouse RNA.

Including BC003355, we found 86 regions of high, unpredictable coverage (hunc) spread across 65 transcripts (Additional file 6). Therefore, over 6% of the 963 IVT transcripts contained regions showing wild but reproducible variations in RNA-seq coverage between samples. While identifying these hunc regions, we used a two-stage filter to eliminate variable regions resulting from mouse reads mapped to highly similar human sequences. First, we eliminated all hunc regions coming from transcripts with FPKM ≥5 in either mouse-only dataset. Next, to account for localized misalignment of mouse reads, we filtered out all hunc regions with an average coverage ≥10 in either mouse-only dataset. We also removed those hunc regions with mouse-only coverage ≥10 in the flanking 100 base pairs (bp) on either side. Given the stringent criteria we used to identify these hunc regions (see *Materials and methods* section for full details), it is likely that this is an underestimate. To address the possibility that mouse RNAs may interact with homologous human RNAs and interfere with them in *trans*, we assayed the sequences surrounding these regions using the MEME Suite [18], but we found no sequence motifs these regions have in common. Furthermore, the depth of coverage at these regions did not follow a linear relationship with the increasing mouse RNA, which suggests it is not simply a direct interaction with the background RNA. There is no clear cause for these hunc regions, particularly since we prepared all samples from the same pool of IVT RNA and the only difference between samples was the relative ratios of IVT RNA to mouse liver RNA. We also searched for hunc regions that were divergent between the two replicates, but found none. If such regions do exist, they could be identified and overcome by creating libraries in duplicate. The hunc regions we identified above with expression patterns maintained between replicates present a greater challenge, as they could not be identified and filtered out by creating duplicate libraries. This is particularly problematic for using exon-level expression values to identify alternative splicing events or differential expression. The within-transcript and between-sample variation we see in our IVT-seq data suggests that library generation introduces strong technical biases, which could confound attempts to study the underlying biology.

### Sources of variability in RNA-seq coverage

There are three potential sources for technical bias in library preparation: RNA-specific molecular biology (RNA fragmentation, reverse-transcription), RNA selection method (rRNA depletion, polyA selection), and sequencing-specific molecular biology (adapter ligation, library enrichment, bridge PCR). To identify biases introduced solely by sequencing-specific molecular biology, we created a DNA-seq library from the same MGC plasmids used as templates for the IVT-seq libraries (Additional file 7: Figure S5). In doing this, we skipped the steps specific to the IVT or RNA molecular biology. We also prepared two additional IVT-seq libraries using polyA selection or no selection, instead of rRNA depletion. By comparing our plasmid library data and the IVT-seq data using various selection methods, we could identify which coverage patterns were the result of RNA-specific molecular biology, the RNA selection method, or of some common aspect of the library-generation protocol.

We sequenced the plasmid library using an Illumina MiSeq and aligned the resulting data to the human reference genome using the same method as the IVT-seq libraries. In this plasmid data, we saw 924 of the cDNA clone sequences with FPKM values ≥5, compared to approximately 870 in both of the IVT only samples (Table 1). This small drop in coverage was likely because the IVT RNA goes through more pooling steps during library construction than the plasmids. Furthermore, the plasmids are not affected by transcription and reverse transcription efficiencies. Additionally, the plasmid data mapped to the cDNA sequences with an average, normalized coverage of 42.08, which is within the range of coverage values we see for the IVT-seq samples. We sequenced the no selection and polyA selection libraries on a HiSeq 2500. These data also show cDNA clone coverage values similar to the other IVT-seq libraries.

The plasmid data represents the ‘input’ into the IVT reaction and the no selection data represents the closest measure of its direct output. By measuring the 3′/5′ ratio in depth of coverage for each IVT transcript, we could assess the processivity of the SP6 polymerase. In a perfectly processive reaction, this 3′/5′ ratio would be 1, indicating the polymerase did not fall off the cDNA template and lead to the formation of truncated products. The median 3′/5′ ratios for the plasmid and no selection data were 1 and 0.98, respectively, indicating premature termination of the IVT reaction was not a factor in our analyses.

### Effect of different RNA selection methods on coverage patterns

**Figure 4:**
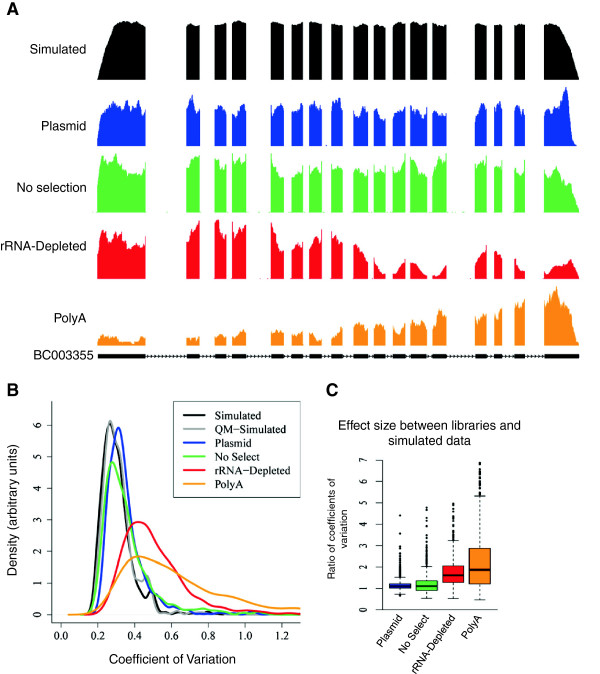
**Sources of bias in RNA-seq coverage. (A)** RNA-seq coverage plots for IVT transcript BC003355 from simulated (black), plasmid (blue), no selection (green), rRNA-depleted (red), and polyA (orange) data. The gene model is displayed in black, below all of the coverage plots. Blocks correspond to exons and lines indicate introns. The chevrons within the intronic lines indicate the direction of transcription. **(B)** Distributions for coefficients of variation across data displayed above, with the addition of the QM-simulated data (gray). Note that while the graph is cutoff at a coefficient of variation of 1.3, the tails for the rRNA-depleted and polyA distributions extend out to 2.13 and 2.7, respectively. **(C)** Effect sizes for the differences in distribution of coefficients of variation between sequencing libraries and simulated data. Effect sizes are calculated as the per-transcript ratios of coefficients of variation between a given library and the simulated dataset. QM, quantity matched.

Our analysis is illustrated by an examination of the coverage plots for BC003355 across all of our different datasets. The high degree of variability we noted in this gene’s coverage plot from our rRNA-depleted data was absent in the no selection and plasmid data (Figure 4A). While the polyA data also showed fewer peaks and valleys than the rRNA-depleted total RNA-seq data, it was marked by the well-documented 3′ bias. These data suggest that the rRNA depletion step is likely responsible for a large quantity of the observed coverage biases.

To quantify the variability for each selection method, we calculated the coefficient of variation at the single base level in coverage for all IVT transcripts across each of these datasets (Figure 4B). Using a Wilcoxon rank-sum test (plasmid n = 924, no selection n = 870, rRNA-depleted n = 869), we found the rRNA-depleted data had significantly higher variability than the no selection and plasmid data (*P* <2.2e^-16^). Furthermore, the rRNA-depleted and polyA libraries were more than 60% more variable on average than the plasmid library (Figure 4C). This suggests that a significant portion of the observed variability in coverage across transcripts in the IVT-seq data is the result of RNA-specific molecular biology, specifically the RNA selection step. Furthermore, after accounting for bias introduced by the sequences themselves (plasmid data) and bias introduced by the IVT reaction (no selection data), we found that 50% of transcripts had two-fold and 10% had 10-fold variation in within-transcript expression (Table 2 and Additional file 5: Figure S4). While it is well-appreciated that polyA selection introduces bias, we found that rRNA-depleted data introduced just as much if not more. Neither simulated dataset showed transcripts with a two-fold or higher change in within-transcript expression. Again, this suggests that the observed within-transcript variations are not the result of alignment artifacts or sampling due to low or high expression. One commonly acknowledged source of bias arises from random priming during library preparation [10]. When we examined the different libraries, we saw that fragments from all of the RNA-seq data showed nucleotide frequencies characteristic of random priming bias (Additional file 8: Figure S6). As expected, the plasmid data showed no such bias, since it was derived directly from DNA and did not require a cDNA-generation step. However, the significant differences between all RNA libraries suggest that bias from random priming is not the only factor. The plasmid and no selection data still contain a fair amount of variability when viewed alongside the simulated data (Figure 4A; black). When we examined the entire dataset, both the plasmid and no selection data had significantly higher variation than either simulated dataset (Wilcoxon rank-sum test; simulated data n = 963, QM-simulated data n = 869, plasmid n = 924, no selection n = 870; *P* <2.2e^-16^). These data suggest that sequencing-specific molecular biology common to all libraries we prepared (adapter ligation, library amplification via PCR) is also responsible for a portion of the observed coverage variability and sequencing bias.

### Biases associated with sequence features are dependent on RNA selection method

Given these significant differences in coverage variability, we sought to identify sequence features that might contribute to this bias. We considered three quantifiable sequence characteristics: hexamer entropy, GC-content, and sequence similarity to rRNA (see *Materials and methods* for a detailed description of these metrics). For each sequencing strategy (plasmid, no selection, rRNA-depleted, polyA), we tested if any of the three sequence characteristics had a significant effect on variability in sequencing coverage, as measured by the coefficient of variation. While we are primarily focused on coverage variability as an indicator of sequencing bias, we also looked at depth of coverage, as measured by FPKM.

**Figure 5:**
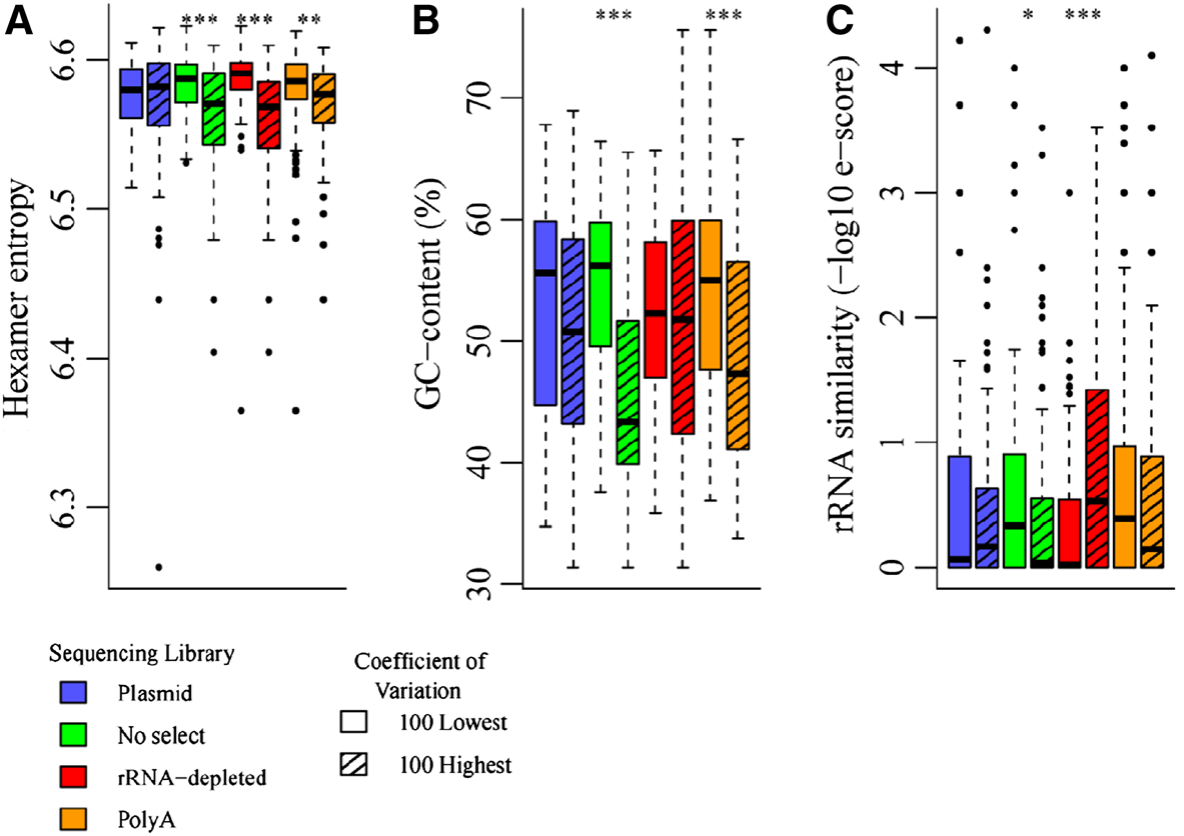
**Effects of sequence characteristics on coverage variability.** Distributions of **(A)** hexamer entropy, **(B)** GC-content, and **(C)** rRNA sequence similarity for the 100 transcripts with the highest and lowest coefficients of variation for transcript coverage from the plasmid, no selection, rRNA-depleted, and polyA libraries. Asterisks indicate the significance of a Wilcoxon signed-rank test comparing values for the listed sequence characteristics between each pair of groups from the same sample. *P <0.05; **P <0.01; ***P <0.001.

For each sequencing strategy, we sorted the transcripts by coverage variability or depth. Next, we selected the 100 most and 100 least extreme transcripts from each list. We compared the values of the sequence characteristics between the 100 most and 100 least extreme transcripts using a Wilcoxon rank-sum test. Significant *P*-values indicate a significant association of the sequence characteristic with coverage variability and/or depth. The results of our analysis are displayed as box-plots (Figure 5 and Additional file 9: Figure S7).

To check for any confounding effects between coverage depth and variability, we tested the least and most expressed transcripts for any correlations with variability in coverage (Additional file 10: Figure S8). The polyA library showed a significant correlation (*P* <2.2e^-16^) between coverage variability and depth, which indicates sequence features could be affecting coverage through variability (or vice versa). The rRNA-depleted data showed a slight, significant correlation (*P* = 0.04933). It is possible some feature of RNA selection affects both variability and coverage, given that we saw no significant correlations for the two remaining samples. This indicates that coverage variability and depth are independent for the plasmid and no selection data.

All three sequence characteristics had a significant association with variability and depth-of-coverage in at least one of the sequencing strategies. In particular, lower hexamer entropy, a measure of sequence complexity [19-21], was strongly associated with higher variance in all of the RNA libraries (no selection *P* = 4.712e^-05^; rRNA depletion *P* = 3.956e^-11^; polyA *P* = 0.003921; Figure 5A). This suggests that bias associated with hexamer entropy is due partially to RNA-specific procedures in library preparation. Furthermore, an association with lower hexamer entropy indicates there are more repeat sequences in the transcripts with higher variability. This could be indicative of complex RNA secondary structures, as repeated motifs could facilitate hairpin formation. Furthermore, the absence of this association from the plasmid data suggests that this observation was not due to mapping artifacts. The plasmid data contained the same sequences as the RNA-seq data, and would be subject to the same biases introduced by our exclusion of multi-mapped reads.

Higher GC-content was strongly associated with lower coverage variability in the no selection and polyA data (*P* = 5.627e^-13^; *P* = 4.914e^-05^; Figure 5B), suggesting that the effects of GC-bias on within-transcript variability could arise, in part, due to some RNA-specific aspects of library preparation. Also, it appears that GC-bias was not a significant contributing factor to either depth of coverage or the extreme variability in the rRNA-depleted data. Meanwhile, lower GC-content was associated with higher coverage in the plasmid data (*P* = 3.776e^-05^), and lower coverage depth in the no selection and polyA libraries (no selection *P* = 8.531e^-05^; polyA *P* = 0.0009675; Additional file 9: Figure S7B). Given that this trend switched directions between the plasmid library and the RNA libraries, this also suggests that some RNA-specific aspect of library preparation is introducing GC-bias distinct from the high GC-bias associated with Illumina sequencing [22].

Interestingly, higher rRNA sequence similarity was associated with higher coverage variability in the rRNA-depleted library (*P* = 9.006e^-05^) and lower variability in the no selection library (*P* = 0.0367; Figure 5C). It is unsurprising that similarity to rRNA sequences contributed to variability in the rRNA-depleted data, given that rRNA depletion is based upon pair-binding between probes and rRNA sequences. While it is unclear why this trend was reversed in the no selection library, it is striking given the significant increase in within-transcript variability between the no selection and rRNA-depleted libraries (Figure 4B). Furthermore, we saw a slight but highly significant correlation (Pearson R^2^ = 0.308452; *P* <2.2e^-16^) between a transcript sequence’s similarity to rRNA and the magnitude of the difference in coverage between the no selection and rRNA-depleted libraries (Additional file 11: Figure S9 and Additional file 12). While the majority of the factors contributing to the extreme bias in sequence coverage we saw in the rRNA-depleted data remain unclear, our data suggest this could be partially due to depletion of sequences homologous to rRNA.

Taken together, our data demonstrate the utility and potential of the IVT-seq method to identify sources of technical bias introduced by sequencing platforms and library preparation protocols.

## Conclusions

In this study, we present IVT-seq as a method for assessing the technical variability of RNA-seq technologies and platforms. We created a pool of IVT RNAs from a collection of full-length human cDNAs, followed by high-throughput sequencing (Figure 1). Because we know the identities and sequences of these IVT transcripts, and because they were created under conditions not affected by splicing and post-transcriptional modification, they are ideal for identifying technical biases introduced during RNA-seq library generation and sequencing. We used this method to demonstrate that library generation introduces significant biases in RNA-seq data, adding extreme variability to coverage and read-depth along the length of sequenced transcripts (Figure 2). Our most striking finding was that over 50% of the IVT transcripts showed more than two-fold differences in this within-transcript coverage attributable to library preparation and sequencing in the polyA and rRNA-depleted data (Table 2). We prepared all RNA-seq libraries from the same pool of IVT RNA, so these differences were due to library construction and sequencing methods. Furthermore, 6% of the IVT transcripts contained hunc regions with variable coverage across different dilutions of IVT and mouse liver RNA (Figure 3). We found it particularly concerning that these huncs were consistent between replicates, as this means these regions cannot be identified and avoided by making replicate libraries. While the exact cause of this effect is unclear, it could be due to some *trans* interaction between different RNA (human IVT RNA and the background mouse liver RNA). If this is the case, it could prove difficult to account for, given the challenges we have already encountered making predictions for miRNA targets and RNA secondary structure. Based on these results, we strongly recommend caution in interpreting exon-level quantification data, particularly for identifying and quantifying alternative splicing events, without further understanding of these biases.

Using simulated data and by sequencing at various stages of the process (plasmids, unselected IVT RNAs, rRNA-depleted, and polyA selected), we found each step introduced bias. Regions of certain IVT transcripts were under-represented in both DNA and RNA, suggesting something inherent in their structure may resist cloning and sequencing properly. The IVT reaction had its own biases; however, by and large, it worked extremely efficiently with 90% of the input templates producing transcripts at detectable levels. PolyA sequencing revealed the well-described 3′ bias. Finally, we saw extreme bias introduced by the rRNA depletion step. Though we have yet to find the majority of the sources for this extreme bias, knowing that it occurs and that it is at least partially due to rRNA sequence similarity is an important first step. By making this data available to the community, we hope that new experimental and analysis methods can be developed to account for the biases inherent in various aspects of RNA-seq.

Moreover, IVT-seq could be more broadly employed. By itself, the MGC collection has cDNAs derived from more than 16,000 mouse and human genes, including hundreds of genes for which there are more than one form. Therefore, in principle, it is possible to generate sequence profiles for representatives for nearly two thirds of the mammalian transcriptome, or spike in datasets to develop new and better methods for splice form detection and quantification. Similar profiling approaches could do the same for other organisms. In addition, IVT-seq is also immediately relevant to RNA-seq method development, for example, developing new protocols or refining existing ones. Finally, the method is not specific to Illumina sequencing and could be used to account for bias in other sequencing chemistries and methods (for example, SOLiD, Ion Torrent, PacBio).

Importantly, we are not suggesting that current generation RNA-seq is not a fantastic new technology or that quantification data is incorrect, particularly given the validated, reproducible results researchers have been able to gain through its use. Rather, we wish to provide a cautionary note that our understanding of this technology is still relatively new and incomplete. It is our hope that through the use of this data and IVT-seq, we will develop the means to minimize or account for bias in RNA-seq and truly realize the vision of digital gene expression.

## Materials and methods

### Amplification of plasmid library

Glycerol stocks containing individual cDNAs (cloned into pCMV-Sport 6 plasmid) from the MGC [17] were produced. Plasmid DNA was extracted from these glycerol stocks and plated at 50 ng per well in 384-well plates. The contents of three 384-well plates (total of 1,062 human transcripts; Additional file 1) were collected as follows: 10 μl sterile dH_2_O was added to each well and incubated at 37°C for 10 min to resuspend plasmid DNA in water. Plasmid DNAs were collected and combined in 1.5 mL tubes with the aid of a multichannel pipette and concentrated by ethanol precipitation. To amplify the library, 10 ng of plasmid library was transferred into *E. coli* DH5α cells (Invitrogen, Life Technologies, Carlsbad, CA, USA, catalog no. 18258–012). The heat shock method was used to transform *E. coli*. Briefly, cells were incubated with plasmid library for 5 min on ice and were subjected to 42°C for 30 s. Cells were transferred back to ice and incubated for 2 min. Next, 0.95 mL S.O.C. medium (Invitrogen, catalog no. 15544-034) was added to the cells before incubation at 37°C for 1 h with shaking at 225 rpm. Cells were plated on LB-agar (Thermo Fisher Scientific, Waltham, MA, USA, catalog no. BP9724-500) plates containing 100 μg/ml ampicillin. Plates were incubated for 16 h at 37°C to grow the colonies and 3,500 (approximately three times the library size) colonies were collected with liquid LB (Thermo Fisher Scientific, catalog no. BP9723-500). Cells were transferred into 100 mL liquid LB and incubated at 37°C for 2 h. Plasmids were purified using Qiagen (Hilden, Germany) maxiprep kit (catalog no. 12163), according to the manufacturer’s protocol.

### *In vitro* transcription from plasmid library

Plasmids were linearized by NotI enzyme so that the SP6 polymerase promoter site was upstream of the sequences to be transcribed. Reactions consisted of 5 U NotI (New England Biolabs, Ipswich, MA, USA, catalog no. R3189L), 5 μg library plasmid DNA, 1 X NEBuffer 4 (supplied with enzyme), and 90 μl of dH_2_O. The reaction was incubated at 37°C for 2 h to achieve complete digestion. We assessed the complete digestion of plasmid DNA using DNA gel electrophoresis. To eliminate NotI and possible RNase in reaction mixture, the sample was subjected to Proteinase K treatment. SDS and Proteinase K were added to the reaction mixture to a final concentration of 0.5% and 100 μg/mL, respectively. The sample was incubated at 37°C for 30 min. After Proteinase K treatment, the sample was subjected to phenol/chloroform extraction, followed by ethanol precipitation. The pellet was dissolved in 50 μl of RNase-free water. Next, *in vitro* transcription was carried out using MAXIscript® SP6 Kit (Ambion, Life Technologies, catalog no: AM1308). The reaction was composed of 1 μg of library plasmid, 1X transcription buffer, 0.5 mM of nucleoside triphosphates (GTP, ATP, CTP, and UTP), 40 U of SP6 RNA polymerase, and 10 μl of RNase-free water. The reaction was incubated at 37°C for 30 min. Next, the sample was treated with TURBO DNase to remove the plasmid templates. Briefly, 10 U of TURBO DNase (included with the MAXIscript SP6 Kit) were added to the reaction mixture and incubated at 37°C for 15 min. To stop the reaction, 1 μL of 0.5 M EDTA was added. To remove unincorporated nucleoside triphosphates and other impurities, the sample was precipitated with ammonium acetate/ethanol. The following reagents were added to the DNase -treated reaction mixture: 30 μL RNase-free water to bring the volume to 50 μL, 5 μL of 5 M ammonium acetate, and three volumes of 100% ethanol. The sample was chilled at -20°C for 30 min and then centrifuged at maximum speed in a 4°C table-top microcentrifuge. The supernatant was discarded and the pellet washed with ice-cold 70% ethanol. The pellet was dissolved in 50 μL of RNase-free water and the quality of RNA was assessed by agarose gel electrophoresis. In addition, PCR was carried out with IVT RNA to confirm total depletion of plasmid DNA.

### Mouse liver collection and RNA extraction

Wild-type, six-week old male C57/BL6 mice were acquired from Jackson Laboratories (Bar Harbor, Maine, USA). Mice were sacrificed and liver samples were quickly dissected and snap-frozen in liquid nitrogen. RNA was isolated from frozen mouse liver samples by TRIzol reagent according to the manufacturer’s protocol (Invitrogen, catalog no. 15596–026). All animal experiments were performed in accordance with the approval of the Institutional Animal Care and Use Committee.

### Construction and sequencing of RNA-seq library from IVT RNA

IVT RNA (2,500 ng, 150 ng, 75 ng, 15 ng, and 0 ng) was pooled with mouse liver RNA (0 ng, 2,350 ng, 2,425 ng, 2,485 ng, and 2,500 ng respectively) to a final quantity of 2.5 μg. Each pool was split into two replicate samples of 1 μg each. RNA pools were treated with Ribo-Zero Gold Kit (Epicentre, Illumina, San Diego, CA, USA, catalog no. RZHM11106) and converted into Illumina RNA-seq libraries with the TruSeq RNA Sample Preparation Kit (Ilumina, catalog no. FC-122-1001). Briefly, rRNA was removed from 1 ug of pooled RNA using Ribo-Zero Gold Kit and purified via ethanol/sodium acetate precipitation according to the manufacturer’s protocol. After drying, the RNA pellet was dissolved in 18 μL of Elute, Prime, Fragment mix (provided with the TruSeq RNA Sample Preparation Kit). RNA was fragmented for 8 minutes and 17 uL of this fragmented RNA was used to make the RNA-seq library according to Illumina TruSeq RNA Sample Preparation Kit protocol. After fragmentation and priming, first strand cDNA synthesis with SuperScript II (Invitrogen, catalog no. 18064014), second-strand synthesis, end-repair, a-tailing, and adapter ligation, the library fragments were enriched with 15 cycles of PCR. The quality and size of the library was assessed using Agilent (Santa Clara, CA, USA) 2100 BioAnalyzer. The five libraries from each replicate were pooled together and sequenced using a single lane from an Illumina HiSeq 2000 (paired 100 bp reads).

### Construction and sequencing of plasmid library

MGC plasmids were linearized by NotI-HF enzyme as before. These linearized plasmids were then fragmented using a Covaris (Woburn, MA, USA) S220 Focused-ultrasonicator. Briefly, 1.2 μg of linearized plasmid in a final volume of 60 uL of H_2_O was loaded into a microTUBE (Covaris, catalog no. 520045). The ultrasonicator was de-gassed and prepared according to the manufacturer’s protocol. Linearized plasmids were sonicated using the following conditions: intensity 5, duty factor 10%, cycles per burst 200, time 120 s, and water bath temperature 7°C. Fragmented plasmids were gel-purified using a 1% agarose gel (BioRad, Hercules, CA, USA, catalog no. 161–3107) and TAE running buffer (BioRad, catalog no. 161–0743). A slice between 100 bp and 700 bp was excised from this gel. DNA was purified from this gel slice using the MinElute Gel Extraction Kit (Qiagen, catalog no. 28606) according to the manufacturer’s protocol. Fragmented DNA was converted into a sequencing library using the TruSeq DNA Sample Preparation Kit (Illumina, catalog no. FC-121-2001). End repair, adenylation, adapter ligation, gel size-selection, and PCR enrichment were performed according to the manufacturer’s protocol. During the gel size-selection, a band between 300 bp and 500 bp was excised. The quality and size of the library was assessed using Agilent 2100 BioAnalyzer. This library was sequenced using an Illumina MiSeq (paired 100 bp reads).

### Construction and sequencing of no selection and polyA libraries

As with the other RNA-seq libraries, these libraries were prepared using the TruSeq RNA Sample Preparation Kit (Illumina, catalog no. FC-122-1001). For the polyA sample, 1 μg of IVT RNA was treated with polyA selection reagents included with the TruSeq RNA Sample Preparation Kit according to the manufacturer’s protocol. The remainder of the library preparation was carried out using the same conditions as for the other IVT RNA samples. For the no selection sample, 100 ng of IVT RNA at a concentration of 100 ng/μL was diluted with 17 μL of Elute, Prime, Fragment mix (provided with the TruSeq RNA Sample Preparation Kit). Again, the remainder of the library preparation was carried out as with the other samples. These samples were sequenced in a single Illumina HiSeq 2500 lane (paired 100 bp reads).

### Aligning, quantifying, and visualizing sequencing data

Raw reads from all sequencing samples were aligned to the human genome (GRCh37/hg19) using the RNA-seq Unified Mapper [14] (RUM; v2.0.4) with default parameters. Mapping statistics for all libraries are included in Additional file 13. RUM also generated RNA-seq coverage plots in bedgraph format, and calculated transcript- and exon-level FPKM values for each IVT transcript (accession numbers listed in Additional file 1). All analyses were performed using uniquely aligned reads (no multi-mappers) from the RUM_Unique and RUM_Unique.cov output files. Quantification was performed using annotations for the IVT transcripts that we downloaded from the MGC Genes track [17] on the UCSC Genome Browser [23]. Those IVT transcripts mapping to multiple loci or overlapping other IVT transcripts were removed from further analysis (marked with an asterisk in Additional file 1). All coverage plots in this paper were visualized in and captured from the UCSC Genome Browser. All statistical tests and correlation plots were performed in R.

### Generating simulated data

Simulated data was generated using the BEERS software package [24] from gene models for IVT transcripts, with an average coverage depth of 1,000 reads (10,000,000 reads total). All error, intronic read, and polymorphism parameters were set to zero. The remaining parameters used default values. For the QM-simulated data, FPKM values from replicate one of the IVT-only data were used as seeds for generating expression levels (40,000,000 reads total). This generated simulated data with FPKM values closely matching those from the real data (Additional file 3: Figure S2B). All other parameters were the same as for the other simulated data.

### Processivity analysis

Coverage data for each IVT transcript was extracted from coverage files for the plasmid and no selection samples. For each transcript, base pair-level coverage data was extracted from the regions spanning 5% to 15% and 85% to 95% of the transcript, by length. For example, given a 1,000 bp transcript, the first region spanned base pairs 50 to 150, and the second region spanned base pairs 850 to 950. These two coverage regions represent the 5′ end and 3′ end of the transcript, respectively. The first and last 5% of the transcript was excluded to avoid artifacts from the fragmentation process. Processivity of each transcript was assessed by the ratio of the mean depth of coverage from both of these regions (3′ region mean/5′ region mean). These processivity ratios were calculated for all transcripts in the plasmid and no selection data, with expression >5 FPKM.

### Calculating fold-change difference in within-transcript coverage

Coverage data for each of the IVT transcripts was extracted from the coverage files for the IVT-only, polyA, and no selection samples. The first and last 200 bp were trimmed from each transcript to prevent edge effects from interfering with the calculations. Due to this trimming, all IVT transcripts with less than 500 bp were discarded. All IVT transcripts expressed with FPKM <5 in any of the samples were discarded from further analysis. Nucleotide-level coverage data was grouped into percentiles based on depth of coverage. Average coverage was calculated across the 10th percentile and the 90th percentile. Fold-change differences in within-transcript coverage were calculated by dividing the 90th percentile average by the 10th percentile average. The list of transcripts with associated fold-change values is included in Additional file 14.

### Identifying hunc regions

Coverage data for each of the IVT transcripts was extracted from the coverage files from each of the rRNA-depleted datasets (replicate dilution series: IVT-only, 1 IVT:1 mouse, 1 IVT:2 mouse, 1 IVT:10 mouse, and mouse-only). These coverage plots were normalized between zero and one to allow comparison between different dilutions. For each nucleotide position in a transcript, the deviation in coverage between each of the samples was calculated using the median absolute deviation (MAD), due to its resistance to outliers. MAD scores were calculated across the different dilutions using R’s *mad* function with constant = 1. Next, a sliding window was used to calculate the average MAD in the 100 bp windows centered on each nucleotide in the transcript. The first 300 and last 250 windows were trimmed from each transcript to avoid confounding variability due to edge effects or fragmentation artifacts. All analysis up until this point was carried out separately on the two replicate datasets. The 95th percentile of MAD scores was calculated for each of the replicates using R’s *quantile* function (replicate 1: 0.08810424; replicate 2: 0.07183765). Only those regions with at least 20 contiguous windows having MAD scores above the appropriate 95th percentile values were retained for further analysis. Next, the BEDTools [25] intersect function was used to remove any regions with high MAD scores not present in both replicates. Finally, these remaining regions of high coverage variability were filtered for mouse reads misaligned to the human reference. Any regions coming from transcripts with FPKM ≥5 in the mouse-only samples were discarded. To account for localized misalignment of mouse reads, any regions with an average coverage >10 in the mouse-only samples or in the 100 bp on either side of the region were discarded. These remaining regions comprise the final list of regions with high coverage variability. To search for hunc regions not maintained between replicates, windowed MAD scores from replicate two were subtracted from those of replicate one. The 2.5th and 97.5th percentiles of these difference values were used as cutoffs (2.5th percentile: −0.07053690; 97.5th percentile: 0.09134876) to pull out the most extreme positive and negative difference values. Regions corresponding to these extreme difference values were filtered as above. Additionally, those difference regions within 200 bp of a previously identified hunc region were filtered out. This last filtering step accounted for cases where a difference region with high MAD scores was just an extension of an existing hunc region. Hunc regions and difference regions were manually checked to determine whether they represented regions where expression patterns deviated from the remainder of the transcript.

### Generating sequence characteristics

Sequences for each transcript were collected in R using the BSgenome, GenomicRanges, and GenomicFeatures packages. Hexamer entropy for each transcript was calculated as follows: occurrences of all possible hexamers in a given transcript were counted. These counts were converted into frequency space, and these frequency values were used to calculate the Shannon entropy. Shannon entropy is commonly used to represent complexity in nucleotide sequences or multiple alignments [19-21]. Similarity of transcripts to rRNA sequences was calculated as follows: each transcript was aligned to 45S (NR_046235.1) and 5S (X71804.1) rRNA using NCBI BLAST [26] and the e-score for the best alignment was saved.

### Sequence characteristic analysis

The list of IVT transcripts was sorted by transcript-level coefficients of variation for each library method (plasmid, no selection, polyA, replicate one of rRNA-depleted IVT-only). All transcripts with transcript-level FPKM ≤5 were excluded from further analysis. From this sorted list, the transcripts with the 100 least and 100 most extreme coefficients of variation were collected for each of the above sequencing samples. The values for hexamer entropy, GC-content, and rRNA sequence similarity were compared between every pair of 100 least and 100 most extreme coefficients of variation using a Wilcoxon signed-rank test (implemented in R as the *wilcox.test* function). This entire analysis was repeated using transcript-level FPKM values instead of the coefficients of variation. All boxplots were prepared using R.

### Data access

We deposited all sequencing data in the NCBI Gene Expression Omnibus under accession number [GEO:GSE50445]. We also loaded the coverage tracks on the UCSC Genome Browser, making them available to the community (comparison between different selection methods [27]; comparison between replicates [28]).

## Abbreviations

BEERS: Benchmarker for Evaluating the Effectiveness of RNA-Seq Software; bp: base pairs; FPKM: fragments per kilobase of exon per million mapped reads; hunc: high, unpredictable coverage; IVT: *in vitro* transcribed; MAD: median absolute deviation; MGC: Mammalian Gene Collection; RUM: RNA-Seq unified mapper.

## Competing interests

The authors declare that they have no competing interests.

## Authors’ contributions

JBH and RI conceived the research. NFL and IHK created the Illumina sequencing libraries. NFL, GRG, MB, RT, and RZ developed and performed the computational analysis. HD and AP contributed code. RI, JK, HD, KH, and AP assisted with the computational analysis. JBH, GRG, and NFL wrote the paper. All authors read and approved the final manuscript.

## Supplementary Material

Additional file 1 Accession numbers for IVT transcripts. Click here for file

Additional file 2: Figure S1 Expression comparison between replicates. **(A)** Correlation plots for log10 transcript-level FPKM values between replicate IVT-seq samples. Pearson R^2^ values for the correlations are included as inserts in each plot. **(B)** Distribution of FPKM values in both replicates of the IVT-only sample. FPKM values are plotted on the x-axis in log10 space. The y-axis is plotted in arbitrary density units. Click here for file

Additional file 3: Figure S2 Expression comparison between simulated and IVT data. Correlation plots for log10 transcript-level FPKM values between **(A)** simulated data or **(B)** QM-simulated data, and replicate one of the IVT-only data. Pearson R^2^ values for the correlations are included as inserts in each plot. Click here for file

Additional file 4: Figure S3 Coverage patterns are reproducible across replicates. Coverage patterns from both replicates for all transcripts in Figure 2. RNA-seq coverage plots from replicate IVT only samples (red – replicate one; blue – replicate two) for **(A)** BC015891, **(B)** BC009037, and **(C)** BC016283 are displayed according to the gene model (green), as it is mapped to the human reference genome. Blocks correspond to exons and lines indicate introns. The chevrons within the intronic lines indicate the direction of transcription. Numbers on y-axis refer to RNA-seq read-depth at a given nucleotide position. All transcripts are displayed in the 5ʹ to 3ʹ direction. Click here for file

Additional file 5: Figure S4 Fold-change in within-transcript coverage across libraries. The cumulative distribution functions for fold-change in within transcript coverage are displayed for the rRNA-depleted (red), polyA (orange), no selection (green), plasmid (blue), QM-simulated (gray), and simulated (black) datasets. Curves toward the left side of the plot indicate fewer genes contain high fold-change differences in coverage. Curves toward the right side of the plot indicate many genes contain high fold-change differences in coverage. The dotted lines indicate the y-axis values for none of the data (0.0) and all of the data (1.0). This plot is focused on the fold-change values between 1 and 10. See the *Materials and methods* section for full details on the fold-change calculations. Click here for file

Additional file 6 List of regions with high coverage variability (hunc regions). Click here for file

Additional file 7: Figure S5 Plasmid sequencing protocol compared to IVT-seq. The protocol for preparing MGC plasmids for DNA-sequencing library generation is displayed alongside the protocol for preparing IVT transcripts for RNA-seq library generation. Both protocols start by linearizing the plasmids. For DNA-sequencing, linearized plasmids are fragmented via Covaris sonication, and the resulting fragments are taken through the TruSeq protocol. For RNA-sequencing, the linearized plasmids are used as templates for an *in vitro* transcription reaction. IVT RNA is then pooled with mouse RNA, rRNA is removed from pool via Ribo-Zero Gold kit, rRNA-depleted pool is fragmented via metal-ion hydrolysis, and fragmented RNA is converted to cDNA via reverse transcription with random-hexamer priming. The resulting cDNA fragments are then taken through the TruSeq protocol. Click here for file

Additional file 8: Figure S6 Nucleotide frequency as a function of read position for sequencing reads at the 5ʹ ends of cDNA fragments. Frequencies are plotted for plasmid, no selection, rRNA-depleted, and polyA datasets. Click here for file

Additional file 9: Figure S7 Effects of sequence characteristics on coverage depth. Distributions of **(A)** hexamer entropy, **(B)** GC-content, and **(C)** rRNA sequence similarity for the 100 transcripts with the highest and lowest transcript-level FPKMs from the plasmid, no selection, rRNA-depleted, and polyA libraries. Asterisks indicate the significance of a Wilcoxon signed-rank test comparing values for the listed sequence characteristics between each pair of groups from the same sample. **P <0.01; ***P <0.001. Click here for file

Additional file 10: Figure S8 Confounding effects between coverage depth and variability. Distributions of transcript-level coefficients of variation for the 100 transcripts with the highest and lowest transcript-level FPKMs from the plasmid, no selection, rRNA-depleted, and polyA libraries. Asterisks indicate the significance of a Wilcoxon signed-rank test comparing values for the listed sequence characteristics between each pair of groups from the same sample. *P <0.05; ***P <0.001. Click here for file

Additional file 11: Figure S9 rRNA sequence similarity and coverage bias in rRNA-depleted data. Correlation plot between Smith-Waterman alignment score to rRNA sequences and the magnitude of the decrease in coverage depth between no selection and rRNA-depleted samples. A coverage drop of 1.0 indicates a large decrease in coverage between the no selection and rRNA-depleted samples. A coverage drop of 0 indicates no difference between the two samples. For full details on this analysis, see Additional file 12. Click here for file

Additional file 12 Description of window analysis of rRNA sequence similarity. Click here for file

Additional file 13 Alignment statistics for all sequencing datasets. Click here for file

Additional file 14 List of transcripts with associated fold-change values in within-transcript coverage. Click here for file
