# Supplementary figures and images for: IVT-seq reveals extreme bias in RNA sequencing
Source: Genome Biol. 2014 Jun 30;15(6):R86. doi: 10.1186/gb-2014-15-6-r86 (PMC4197826; doi:10.1186/gb-2014-15-6-r86)

**Figure S1**

**A**

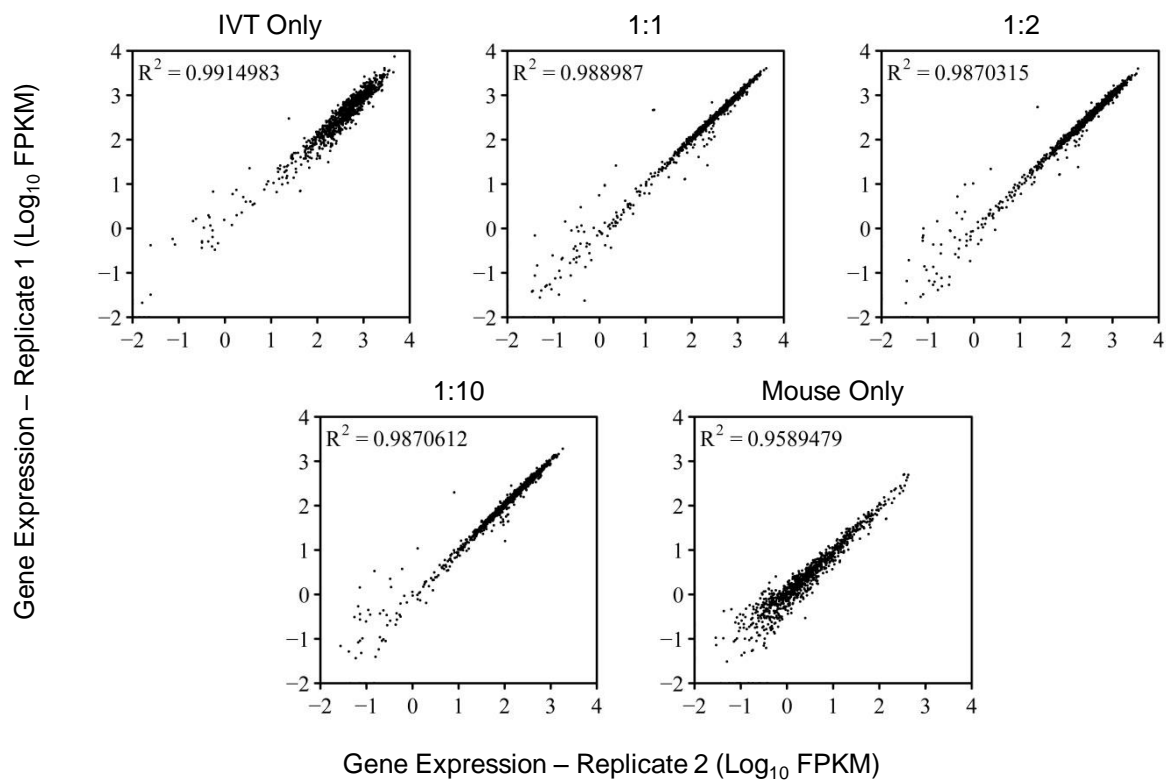

**B**

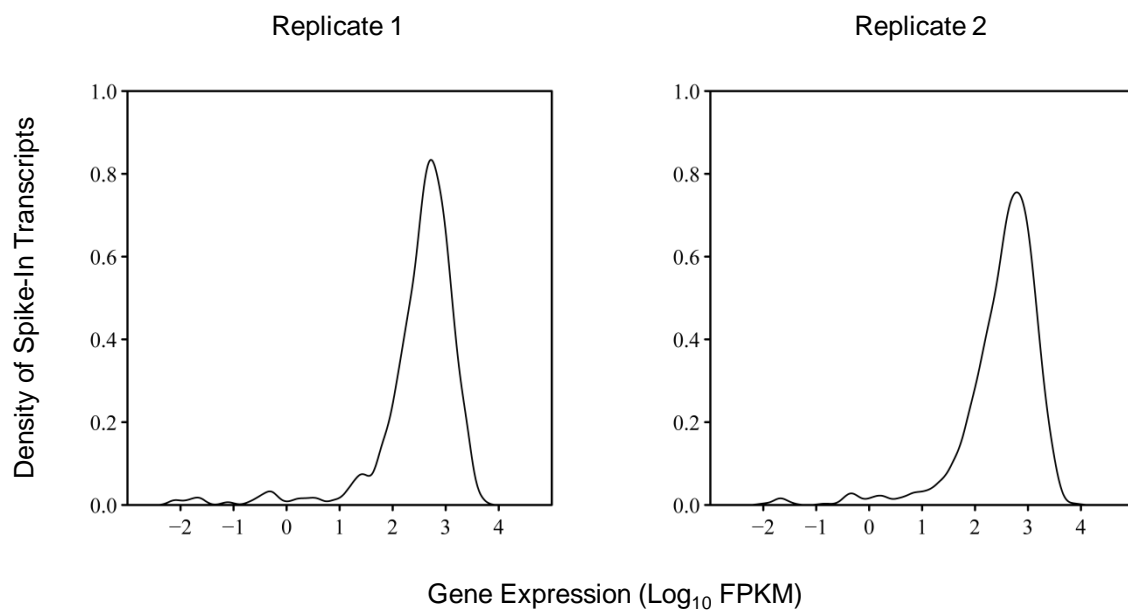

Supplement: Additional file 2: Figure S1 — Expression comparison between replicates. (A) Correlation plots for log10 transcript-level FPKM values between replicate IVT-seq samples. Pearson R2 values for the correlations are included as inserts in each plot. (B) Distribution of FPKM values in both replicates of the IVT-only sample. FPKM values are plotted on the x-axis in log10 space. The y-axis is plotted in arbitrary density units. [file gb-2014-15-6-r86-S2.pdf]

**Figure S2**

**A**

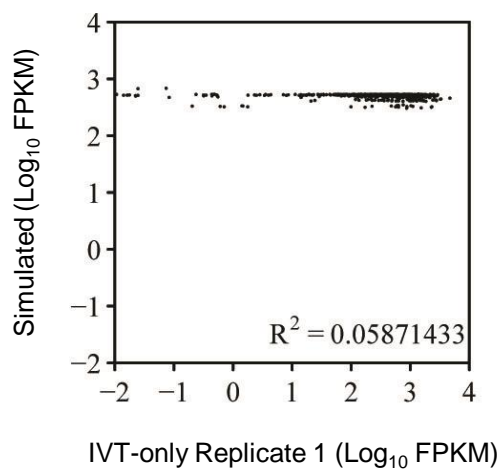

**B**

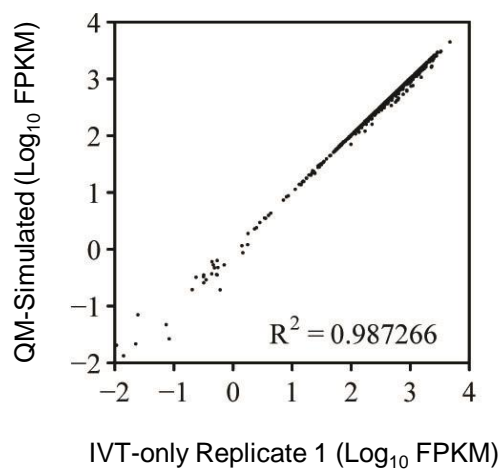

Supplement: Additional file 3: Figure S2 — Expression comparison between simulated and IVT data. Correlation plots for log10 transcript-level FPKM values between (A) simulated data or (B) QM-simulated data, and replicate one of the IVT-only data. Pearson R2 values for the correlations are included as inserts in each plot. [file gb-2014-15-6-r86-S3.pdf]

Figure S3

A

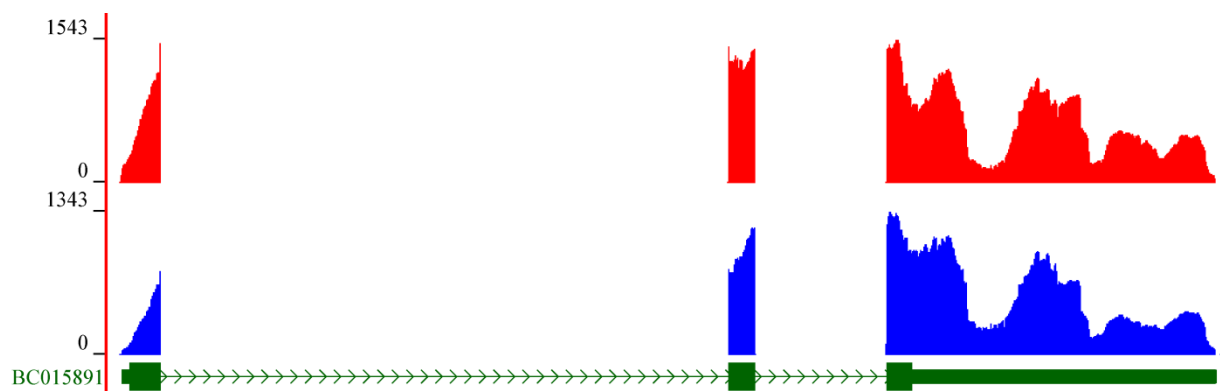

B

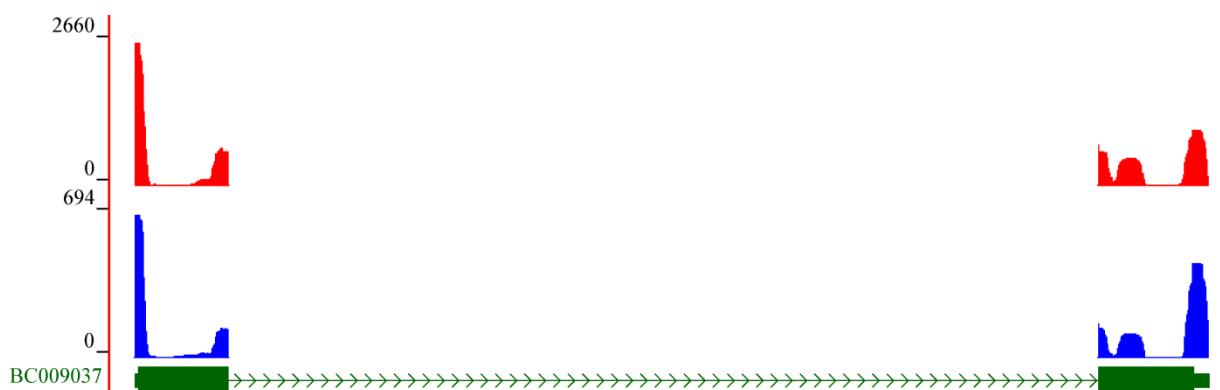

C

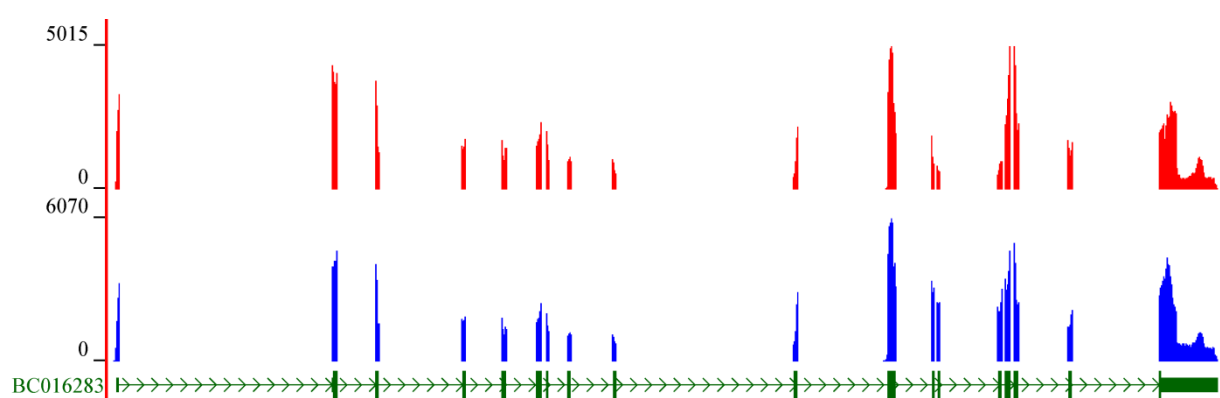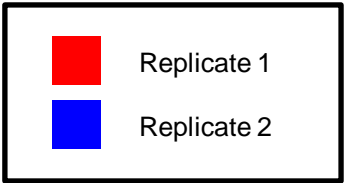

Supplement: Additional file 4: Figure S3 — Coverage patterns are reproducible across replicates. Coverage patterns from both replicates for all transcripts in Figure 2. RNA-seq coverage plots from replicate IVT only samples (red – replicate one; blue – replicate two) for (A) BC015891, (B) BC009037, and (C) BC016283 are displayed according to the gene model (green), as it is mapped to the human reference genome. Blocks correspond to exons and lines indicate introns. The chevrons within the intronic lines indicate the direction of transcription. Numbers on y-axis refer to RNA-seq read-depth at a given nucleotide position. All transcripts are displayed in the 5ʹ to 3ʹ direction. [file gb-2014-15-6-r86-S4.pdf]

**Figure S4**

Within-transcript fold-change in coverage depth

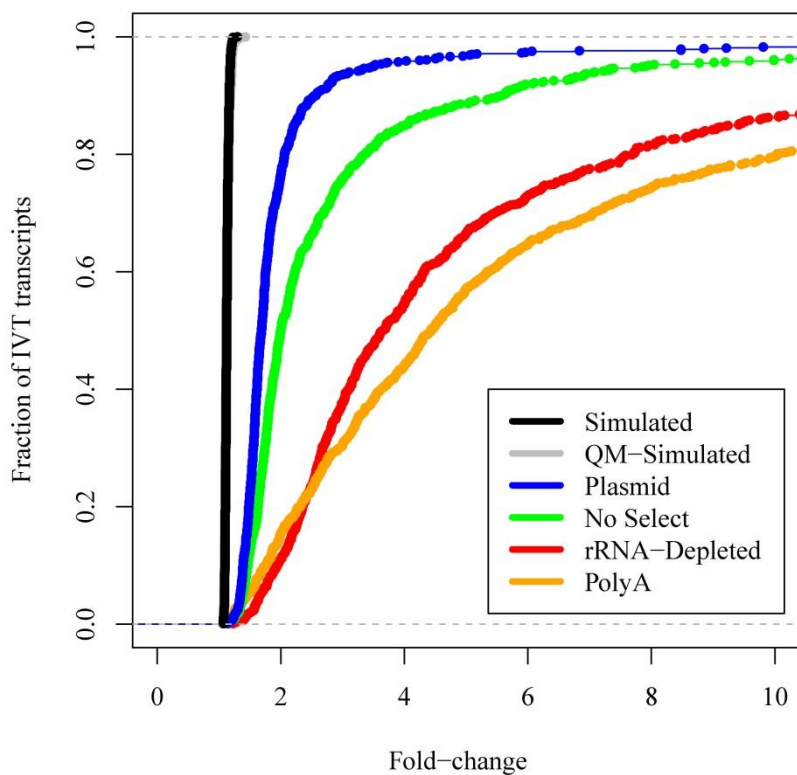

Supplement: Additional file 5: Figure S4 — Fold-change in within-transcript coverage across libraries. The cumulative distribution functions for fold-change in within transcript coverage are displayed for the rRNA-depleted (red), polyA (orange), no selection (green), plasmid (blue), QM-simulated (gray), and simulated (black) datasets. Curves toward the left side of the plot indicate fewer genes contain high fold-change differences in coverage. Curves toward the right side of the plot indicate many genes contain high fold-change differences in coverage. The dotted lines indicate the y-axis values for none of the data (0.0) and all of the data (1.0). This plot is focused on the fold-change values between 1 and 10. See the Materials and methods section for full details on the fold-change calculations. [file gb-2014-15-6-r86-S5.pdf]

Figure S5

MGC Plasmid Library Prep

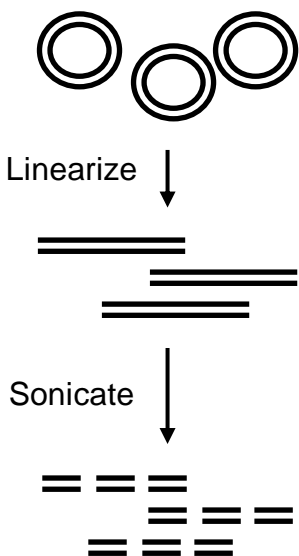

TruSeq  
Protocol

IVT RNA Library Prep

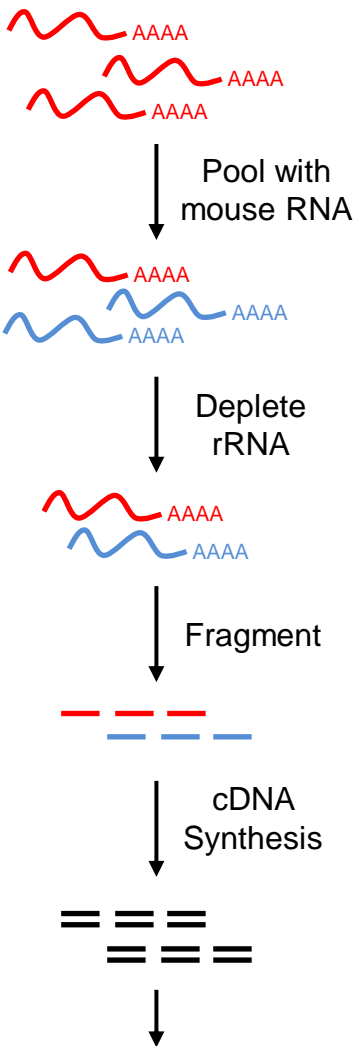

TruSeq  
Protocol

Supplement: Additional file 7: Figure S5 — Plasmid sequencing protocol compared to IVT-seq. The protocol for preparing MGC plasmids for DNA-sequencing library generation is displayed alongside the protocol for preparing IVT transcripts for RNA-seq library generation. Both protocols start by linearizing the plasmids. For DNA-sequencing, linearized plasmids are fragmented via Covaris sonication, and the resulting fragments are taken through the TruSeq protocol. For RNA-sequencing, the linearized plasmids are used as templates for an in vitro transcription reaction. IVT RNA is then pooled with mouse RNA, rRNA is removed from pool via Ribo-Zero Gold kit, rRNA-depleted pool is fragmented via metal-ion hydrolysis, and fragmented RNA is converted to cDNA via reverse transcription with random-hexamer priming. The resulting cDNA fragments are then taken through the TruSeq protocol. [file gb-2014-15-6-r86-S7.pdf]

**Figure S6**

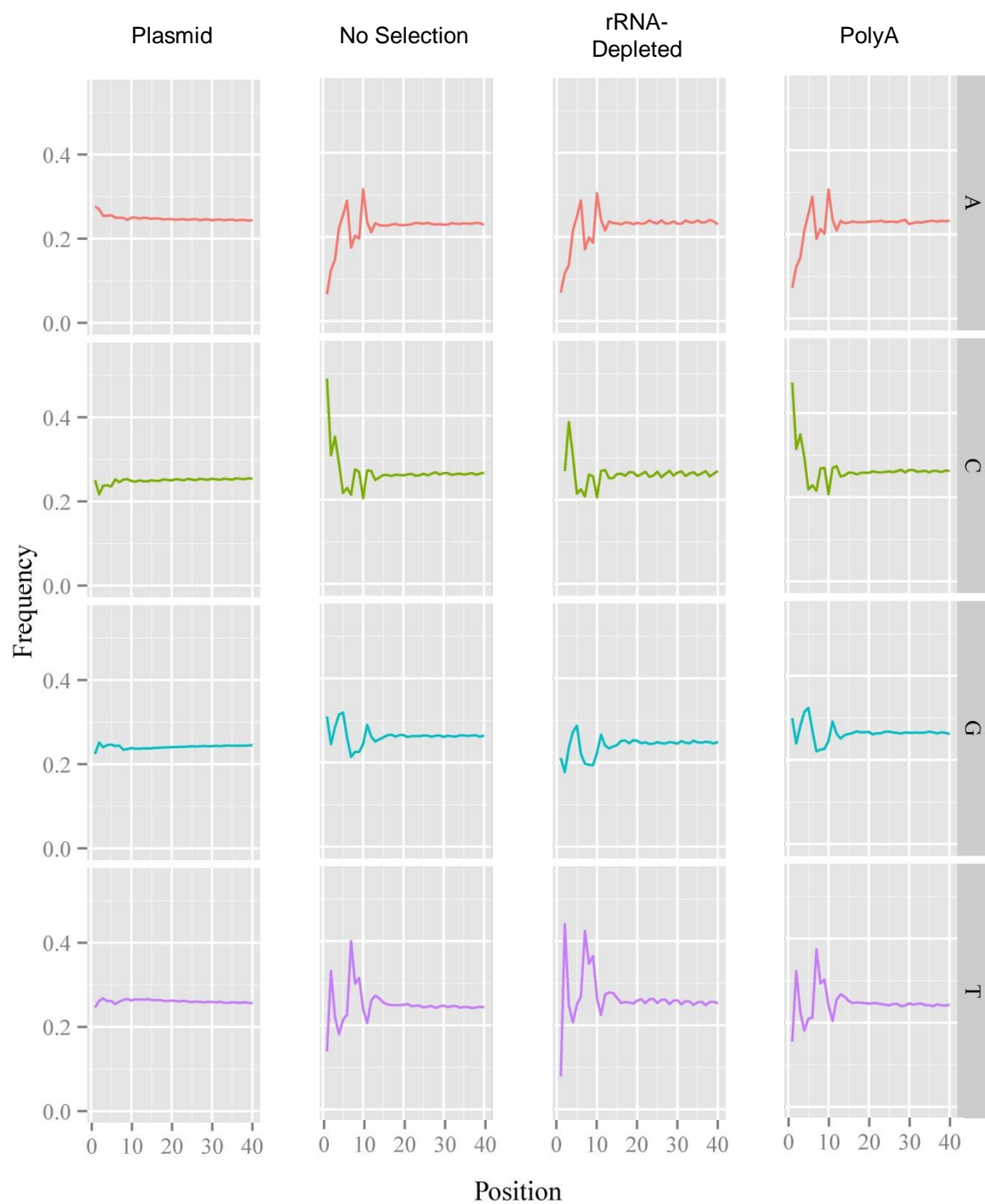

Supplement: Additional file 8: Figure S6 — Nucleotide frequency as a function of read position for sequencing reads at the 5ʹ ends of cDNA fragments. Frequencies are plotted for plasmid, no selection, rRNA-depleted, and polyA datasets. [file gb-2014-15-6-r86-S8.pdf]

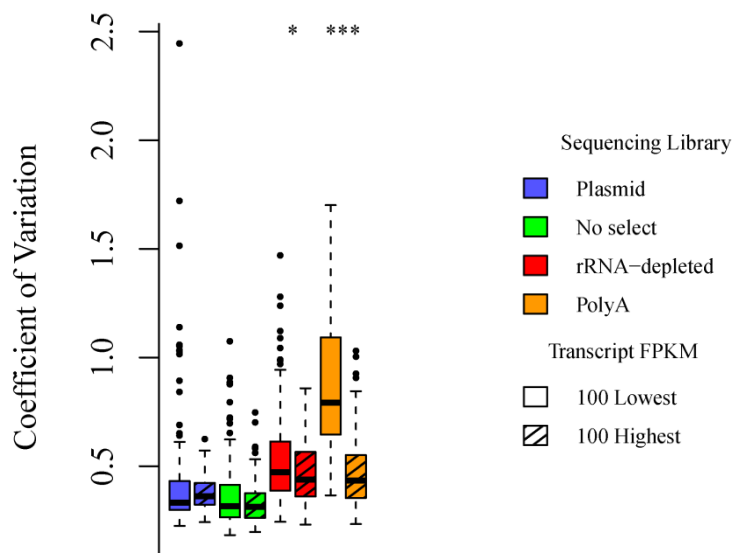

Supplement: Additional file 10: Figure S8 — Confounding effects between coverage depth and variability. Distributions of transcript-level coefficients of variation for the 100 transcripts with the highest and lowest transcript-level FPKMs from the plasmid, no selection, rRNA-depleted, and polyA libraries. Asterisks indicate the significance of a Wilcoxon signed-rank test comparing values for the listed sequence characteristics between each pair of groups from the same sample. *P <0.05; ***P <0.001. [file gb-2014-15-6-r86-S10.pdf]

**Figure S9**

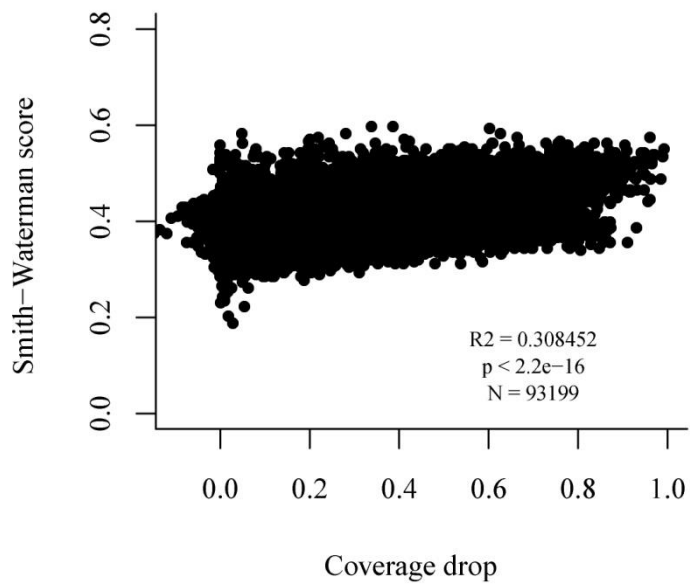

Supplement: Additional file 11: Figure S9 — rRNA sequence similarity and coverage bias in rRNA-depleted data. Correlation plot between Smith-Waterman alignment score to rRNA sequences and the magnitude of the decrease in coverage depth between no selection and rRNA-depleted samples. A coverage drop of 1.0 indicates a large decrease in coverage between the no selection and rRNA-depleted samples. A coverage drop of 0 indicates no difference between the two samples. For full details on this analysis, see Additional file 12. [file gb-2014-15-6-r86-S11.pdf]
