# Supplementary material for: IVT-seq reveals extreme bias in RNA sequencing
Source: Genome Biol. 2014 Jun 30;15(6):R86. doi: 10.1186/gb-2014-15-6-r86 (PMC4197826; doi:10.1186/gb-2014-15-6-r86)
Supplement: Additional file 9: Figure S7 — Effects of sequence characteristics on coverage depth. Distributions of (A) hexamer entropy, (B) GC-content, and (C) rRNA sequence similarity for the 100 transcripts with the highest and lowest transcript-level FPKMs from the plasmid, no selection, rRNA-depleted, and polyA libraries. Asterisks indicate the significance of a Wilcoxon signed-rank test comparing values for the listed sequence characteristics between each pair of groups from the same sample. **P <0.01; ***P <0.001. [file gb-2014-15-6-r86-S9.pdf]

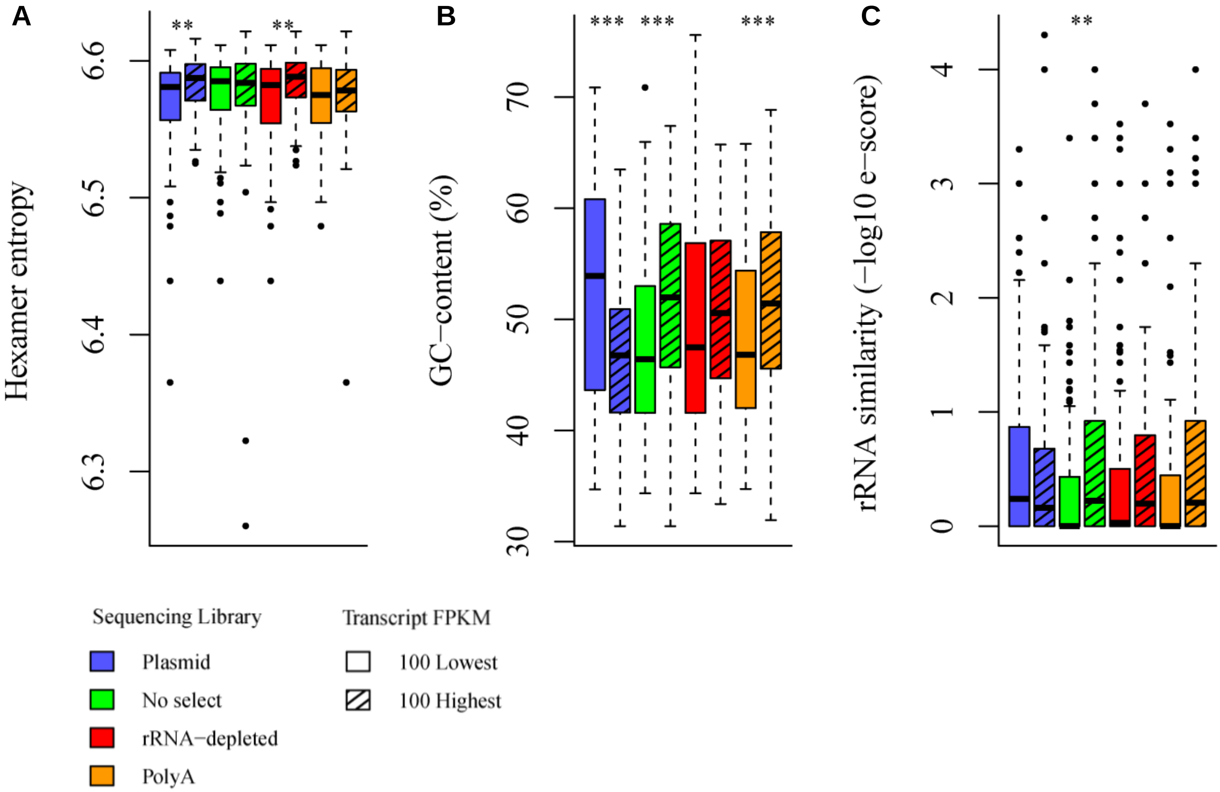

A
Hexamer entropy
6.3
6.4
6.5
6.6
**
**
B
GC−content (%)
30
40
50
60
70
***
***
***
C
rRNA similarity (−log10 e−score)
0
1
2
3
4
**
Sequencing Library
Plasmid
No select
rRNA−depleted
PolyA
Transcript FPKM
100 Lowest
100 Highest
