# Supplementary material for: IVT-seq reveals extreme bias in RNA sequencing
Source: Genome Biol. 2014 Jun 30;15(6):R86. doi: 10.1186/gb-2014-15-6-r86 (PMC4197826; doi:10.1186/gb-2014-15-6-r86)
Supplement: Additional file 12 — Description of window analysis of rRNA sequence similarity. [file gb-2014-15-6-r86-S12.pdf]

Additional file 12: Description of window analysis of rRNA sequence similarity

All transcripts with zero read counts in the no selection and rRNA-depleted data were discarded. Nucleotide-level read counts for each of the remaining transcript were normalized between 0 and 1 as follows: The read depth for each nucleotide was divided by the transcript's maximum read depth. This step was performed to account for any lane effects. Read counts from the rRNA-depleted data were scaled to be strictly less than or equal to their corresponding read counts in the no selection data as follows: ignoring the first and last 100 bp of the transcript, the nucleotide position with the smallest ratio between the no selection and rRNA-depleted data was determined. All nucleotides within that transcript were multiplied by this ratio. This step was performed under the assumption that biases introduced by rRNA-depletion will always result in a loss of coverage relative to the no selection data. A 128 bp window slid across each transcript in 16 bp increments and calculated the following: 1) the highest Smith-Waterman alignment score between the sequence in the current window and a library of rRNA sequences (see below), using the SimMetrics Java package (http://sourceforge.net/projects/simmetrics/). 2) The average difference in coverage between the no selection and rRNA-depleted data. The Pearson correlation between this coverage difference and the Smith-Waterman score in each window was calculated using the cor.test function in R.

Refseq IDs for rRNA sequences used in window analysis of rRNA sequence similarity.

**Refseq_ID**
NR_003286.2
NR_003287.2
NR_023365.1
NR_023366.1
NR_023367.1
NR_023368.1
NR_023369.1
NR_023370.1
NR_023371.1
NR_023372.1
NR_023373.1
NR_023374.1
NR_023375.1
NR_023376.1
NR_023377.1
NR_023378.1
NR_023379.1
NR_046235.1
NR_048572.1
NR_049740.1
